# Enhanced infectivity of bovine viral diarrhoea virus (BVDV) in arginase-producing bovine monocyte-derived macrophages

**DOI:** 10.1080/21505594.2023.2283899

**Published:** 2023-11-15

**Authors:** Lucas José Barone, Nancy Patricia Cardoso, Florencia Celeste Mansilla, Mariángeles Castillo, Alejandra Victoria Capozzo

**Affiliations:** Institute of Virology and Technical Innovations, INTA- CONICET. National Research Council (CONICET), Buenos Aires, Argentina

**Keywords:** Bovine macrophages, arginase activity, pestivirus, azithromycin immunosuppression, replication

## Abstract

Macrophages are important cells of the innate immunity that play a major role in Bovine Viral Diarrhoea Virus (BVDV) pathogenesis. Macrophages are not a homogenous population; they exist in different phenotypes, typically divided into two main categories: classically (pro-inflammatory) and alternatively activated (anti-inflammatory) or M1 and M2, respectively. The role of bovine macrophage phenotypes on BVDV infection is still unclear. This study characterized the interaction between BVDV and monocyte-derived macrophages (Mo-Mφ) collected from healthy cattle and polarized to an M1 or M2 state by using LPS, INF-γ, IL-4, or azithromycin. Arginase activity quantitation was utilized as a marker of the M2 Mo-Mφ spectrum. There was a significant association between arginase activity and the replication rate of BVDV strains of different genotypes and biotypes. Inhibition of arginase activity also reduced BVDV infectivity. Calves treated with azithromycin-induced Mo-Mφ of the M2 state produced high levels of arginase. Interestingly, azithromycin administered *in vivo* increased the susceptibility of macrophages to BVDV infection *ex vivo*. Mo-Mφ from pregnant dams and calves produced higher arginase levels than those from non-pregnant adult animals. The increased infection of arginase-producing alternatively activated bovine macrophages with BVDV supports the need to delve into a possible leading role of M2 macrophages in establishing the immune-suppressive state during BVDV convalescence.

## Introduction

Bovine viral diarrhoea virus (BVDV) is a single-stranded, positive-sense RNA virus [1] that belongs to the *Flaviviridae* family, genus *pestivirus*. BVDV is also classified based on genotypes [2] (BVDV 1, 2 and 3) and biotypes, either cytopathic (cp) or non-cytopathic (ncp), according to the effect on the viability of susceptible cultured cells [3]. The genome encodes for structural and non-structural proteins, and two of them are usually used to assess virus detection, the E2 envelope protein that mediates virus entry to the cells, which varies between strains, and a non-structural protein (NS3) that has helicase/ATPase activity which is highly conserved among pestiviruses. NS3 is only produced when the viral RNA is translated within the infected cell [4] and can be used as a marker of viral infection [5].

BVDV has a worldwide distribution and an important economic impact in the cattle industry [6]. The disease caused by ncp BVDV in cattle has multiple manifestations, which ranges all the way from asymptomatic, through respiratory or gastrointestinal symptoms and up to a persistent infection (PI) that can occur when foetuses are infected during the first trimester of pregnancy. PI animals can undergo a lethal haemorrhagic syndrome [7] when the infecting ncp strain mutates to a cp biotype [8]. Cp strains are self-limited, while ncp strains are of upmost epidemiological relevance.

Acute, self-limiting infections with ncp BVDV are associated with a period of generalized immunosuppression and increased susceptibility to secondary infection [9] with opportunistic pathogens including other viruses and bacteria (i.e. *Mannheimia haemolytica*, *Pasteurella multocida*, SRV, PI3, etc.) [10]. Young animals and pregnant dams are more susceptible to symptomatic BVDV than adult-non-pregnant animals. High genetic variation of BVDV sequence has been observed in pregnant animals, where immunity is already altered because of the pregnancy, allowing a greater number of infecting virus particles to establish a productive infection [11].

There is evidence of impairment of the innate immune system that contributes to the immunosuppressive BVDV convalescence state. BVDV infects a wide variety of cell types but prefers cells of the immune system like macrophages and lymphocytes [12]. We and others demonstrated the BVDV tropism for dendritic cells (DC) with the concomitant temporary impairment of their function. Infection of DC with cp or ncp strain did not induce cell death [13,14], while monocytes are killed by cp strains [13]. Rajput et al. showed that CD14 cells obtained by *ex vivo* 7-d culture of density gradient-isolated monocytes infected with an ncp strain were unable to sustain the formation of infectious virus, although non-structural proteins and viral RNA were found in these cells [15].

Data on infection of macrophages with BVDV are still controversial. Macrophage numbers are greatly increased during BVDV infection in lymph nodes. This increase accompanies massive depletion of lymphocytes in lymph nodes and thymus [16]. Abdelsalam et al. [17] showed that the supernatant of infected macrophages with only virulent BVDV strains induced lymphocyte apoptosis, although the proapoptotic mediators have not been identified, so their role in lymphocyte depletion remains unclear [18,19]. Adler et al. reported that adherent bovine bone marrow-derived macrophages infected with BVDV decreased production of TNF-α. This down-regulation was more relevant for ncp biotypes than for their cp counterparts [20]. Suppression of proinflammatory cytokine expression in response to TLR ligation (except TLR-7) was also described for BVDV-type 2 [21]. Moreover, infection of myeloid with BVDV-type 2 was associated with decreased MyD88 expression [22].

The interaction between BVDV and macrophages is then far from being fully understood and studies performed with macrophages so far do not consider the fact that these cells do not constitute a homogenous population [23]. Macrophages display remarkable plasticity and can change their physiology in response to environmental signals. These changes can produce different populations of cells with distinct functions. A simplified classification is the so-called M1, pro-inflammatory profile, “classically activated” or M2 “alternatively activated,” anti-inflammatory phenotype [24,25]. M1 macrophages produce nitric oxide or reactive oxygen intermediates and participate in host defence, while on the contrary M2 cells are characterized by a high-arginase activity [26], and they conform a spectrum of different variants that go from wound healing to immune regulation. “M2” alternatively activated macrophages have higher arginase activity than those traditionally activated “M1” [27]. It is widely accepted that high arginase activity is a marker of all the M2 spectrum [28].

To the best of our knowledge, the susceptibility of the different phenotypes of macrophages in BVDV infection has not been explored. In this study, we evaluated the interaction between different BVDV strains and monocyte-derived macrophages and demonstrated the association of viral infectivity with arginase-producing, alternatively activated, bovine macrophages.

## Materials and Methods

### Cells

Madin-Darby Bovine Kidney (MDBK) cell line from the American Type Cell Collection (ATCC) was provided by the Institute of Virology (INTA) Argentina. Cells were cultured in (Dulbecco’s Modified Eagle Medium, DMEM) with 10% foetal bovine serum (media and serum were purchased from Gibco, SigmaAldrich, Merck KGaA, Darmstadt, Germany) tested to be free of gamma globulins, mycoplasma, and several viruses including BVDV. Absence of BVDV was confirmed by nested RT-PCR as described before [29] and by serial passages in MDBK cells followed by immunofluorescence using a polyclonal antibody (FITC-goat anti-BVDV conjugate, VMRD).

### Virus

BVDV Singer reference strain was obtained from ATCC. All the other strains are part of INTA’s virus repository and were used before [14,29–33].

BVDV stocks were produced by infecting MDBK cells [32]. Titers were calculated as median 50% tissue culture infective dose (TCID50/ml) [14]. Viral stocks (30 ml each) were stored at −80°C until use. Briefly, MDBK cells were grown in 96-well plates (Corning® Costar® Tewksbury, MA, USA) to a confluence of 80% (10,000 cells per well). Serial four-fold dilutions of the tested samples were made in DMEM and 50 μl added to quadruplicate wells. Plates were incubated for 5 d at 37°C, 5% CO_2_. Then, cells were fixed with 3% PBS-formaldehyde. For cp strains, plates were stained with crystal violet to visualize plaques. When titrating ncp strains, each well was treated with 0.1 M Glycine in PBS followed by 0.2% Triton X-100. The presence of BVDV was revealed with an anti-BVDV polyclonal antiserum conjugated to FITC (VMRD). Infected cells were observed in an inverted fluorescence microscope (Olympus IX71). Titers were calculated as the median tissue 50% culture infective dose per millilitre (TCID50/mL) following the Red and Muench method [34]. Extracellular virus titre from infected macrophages was estimated using the same protocol.

### Animals

Bovines were used mainly as blood donors. We used healthy Aberdeen Angus female cattle or calves depending on the experiment.

The animals selected for all the experiments were always free of BVDV (antigen and antibodies), controlled using a commercial kit (PrioCHECK BVDV PI) and an *in-house* anti-E2 ELISA [30]. They were also free from Bovine Leukemia Virus (BLV), diagnosed with an *in-house* whole BLV viral particle indirect ELISA as described previously [35]. These animals were not vaccinated against BVDV.

Animals did not show any clinical signs at the time of the experiments. Blood was collected by punction of the jugular vein in 50 ml syringes containing 3 ml of 0.1 M EDTA (EDTA was 10% of the total volume). This bleeding protocol has been approved by the National Committee for Care and Use of Experimental Animals (CICUAE) by protocol No. 25/2013 CICUAE – INTA.

For the azithromycin inoculation experiment, six calves under 2 months of age were randomly allocated into two experimental groups of three animals each and named according to the treatment they received: Azithromycin and Not-treated (Control). The dosage and route of administration of azithromycin were used as described by Esteban Turic PhD thesis, 2010 [36]. The animals received one intravenous (IV) azithromycin application (10 mg/Kg) on day 1, followed by 1 g per day orally for the following 3 d. The control group was not intervened. Procedures and sample size calculations followed the national protocols for the Care and Use of Experimental Animals and were approved by CICUAE (Protocol No. 13/2021 CICUAE – INTA)

### Macrophage preparation and stimulation

Peripheral blood was obtained as described above. Peripheral blood mononuclear cells (PBMC) were purified by density gradient centrifugation as described by our group [14] and others [37–39]. Monocytes were obtained from PBMC by selective adherence to multiwell cell plates. Monocytes were cultured for 8–10 d until acquiring the macrophage phenotype using Roswell Park Memorial Institute (RPMI) culture medium supplemented with foetal bovine serum (10%), Hepes (25 mM), and an antibiotic-antimycotic solution (all reagents from Gibco). Mo-Mφ obtained by this method was 99% CD14+ with cell viability above 90% as determined by flow cytometry assays (supplementary file 1, Figure 1).

**Figure 1.**
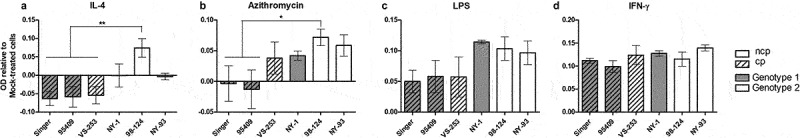
Metabolic activity of macrophages treated with IL-4 (a), azithromycin (b), LPS (c) and IFN-γ (d) and infected with different BVDV strains. XTT reduction assay was used to measure the overall activity of mitochondrial dehydrogenases in the sample that correlates to the amount of formazan formed. Absorbance (OD) values relative to mock- treated cultures are depicted as mean ± SEM. *p < 0.05; **p < 0.01.

To induce the different phenotypes of Mo-Mφ *in vitro*, cells were stimulated by adding 20 ng/ml of LPS (Sigma-Aldrich), 10 ng/ml of recombinant bovine IFN-γ (BioRad), 20 ng/ml of recombinant bovine IL-4 (AbD Serotec-BioRad), or 30 µg/ml of azithromycin [40–42] (Parafarm, Saporiti Pharma, Argentina) to the cell culture media by incubating cells for additional 24 h at 37°C, 5% CO_2_. “M0” Mo-Mφ were mock treated using culture media.

2-(S)-amino-6-boronohexanoic acid (ABH) (Sigma Aldrich, SML 1466) was used as an arginase inhibitor. Mϕ was incubated with 50 μM of ABH [43,44] and treated with INF-γ, IL-4, or azithromycin as described above.

*In vivo* polarization of macrophages towards a high-arginase M2 functional state was performed by treating calves with azithromycin. Six calves under 2 months of age were randomly allocated into two experimental groups of three animals. One group was not-treated (Control), while the other received 10 mg/Kg of azithromycin (Parafarm, Saporiti Pharma, Argentina) IV [36] followed by 1 g of oral azithromycin for the following three consecutive days. Blood and serum samples were taken from all animals prior to applying the treatments (day 0), and on days 5 and 7 post-treatment. At these same time-points, general haematological clinical tests were performed. Mo-Mφ were obtained from peripheral blood on days 5 and 7 post-treatment, and infected or mock-infected *ex vivo* with BVDV 98–124 at a high multiplicity of infection (MOI = 2). Arginase activity and replication (see below) were assessed 24 h post-infection (hpi).

Official animal welfare protocols were approved by the Ethics and Animal Welfare Committee of the Institute of Virology of INTA (25/2013 CICUAE – INTA).

### Cell viability assessment

The metabolic activity of macrophages was measured with CyQUANT™ XTT kit (ThermoFisher, X12223) according to the manufacturer’s instruction. OD values for mock-treated cells were computed as a reference for viable cells. Control dead cells were obtained by incubating the cells overnight (ON) with PBS. Cell-free controls were also included. Percentage of living cells was referred to values of untreated control wells. Samples were run in triplicate.

### NS3 detection

For NS3 determination, a commercial ELISA was used (PrioCHECK BVDV-Ag PI focus; Prionics AG 7,610,140). Cells were harvested, washed, and mixed with the lysis buffer. Total protein content was quantified using the Protein Assay Kit (Pierce BCA; Thermo Scientific, USA). Suspensions containing 0.2 µg of total proteins in a volume of 100 µL were used for NS3 detection by PrioCHECK BVDV-Ag PI focus ELISA following the manufacturer’s instructions. Plates were read in a Multiskan FC (Thermo Scientific) ELISA reader. The results were expressed as OD values (450 nm).

### Arginase activity

Arginase activity was determined on cells using the commercial Arginase Activity Assay Kit (Sigma Aldrich, MAK112) following the manufacturer’s instructions. Briefly, cells were harvested, washed with PBS, and lysed with 10 mM Tris-HCl, pH 7.4, and 0.4% (w/v) Triton X-100 containing complete protease inhibitor cocktail (Santa Cruz Biotechnology). After 10 min of incubation samples were centrifuged at 14,000 G, and supernatants were collected and stored at −80°C until arginase determination. One unit of arginase is the amount of enzyme that converts 1 μmol of L-arginine to urea and ornithine per minute at pH 9.5 at 37 ºC.

### Statistical analysis

The 1- or 2-way ANOVA was used whenever the data obtained complied with a normal distribution (Wilk-Shapiro Normality Test), and their variances were comparable (Bartlett’s Test). In cases in which the number of data was not sufficient to verify the normal distribution of the data, the analysis of the differences was performed using non-parametric methods: Mann-Whitney for comparisons between two groups or Kruskal-Wallis for multiple comparisons, followed by Dunns to determine which groups were different if the results of a Kruskal-Wallis’s test were statistically significant.

To examine the statistical significance of the association between high or low arginase levels and high or low rate of viral replication as categorical variables in a 2 × 2 contingency table with few data, we used Fisher’s exact test. The *p-*value of the test is calculated as if the margins of the table were fixed and therefore provides estimates with the correct number in each category, leading under a null hypothesis of independence to a hypergeometric distribution of numbers in the categories.

The analysis was performed with GraphPad Prism v5.0 (GraphPad Software, CA, USA).

## Results

### *Susceptibility of bovine macrophages to BVDV infection* in vitro

Using arginase quantitation as marker of “M2” alternatively activated macrophage’s profile, we designed an experiment to verify if different treatments influencing arginase activity impacted on the infectivity of six different BVDV strains, including reference strains and local isolates (genotype 1 and 2) of both biotypes (details of the strains have already been published by our group [5]).

Macrophages were prepared by culturing monocytes (Mo-Mφ) obtained from whole blood of four BVDV-free cattle. Mo-Mφ were treated with IFN-γ and LPS to promote the pro-inflammatory profile and IL-4 or azithromycin for anti-inflammatory bias. The rationale behind including azithromycin was to assess a treatment that can be safely used in cattle in future experiments, at an accessible cost. Treated macrophages were then infected with six distinct BVDV strains.

Cp strains induced cell death in IL-4-treated Mo-Mφ that was revealed by a significant decrease (*p* < 0.01) in the capacity of these cells to reduce XTT compared to 98–124 strain (Figure 1a). A significant reduction in cell metabolism (*p* < 0.05) was found in azithromycin-treated Mo-Mφ infected with type-I cp strains (Singer and 95,409) compared to 98–124 ncp strain (Figure 1b). Rounded semi-detached cells were observed after infection with cp strains in both IL-4 and azithromycin-treated Mo-Mφ, while no cytopathic changes were noted for any of the ncp strains (data not shown). Infected macrophages that had been treated with IFN-γ or LPS showed no cp changes and increased their metabolic activity after infection with all the tested strains. No significant differences were found in the OD values between the six strains (*p* > 0.05, Figure 1c and d).

Mo-Mφ treated with IL-4 and LPS had the highest arginase activity (Table 1), followed by azithromycin, while IFN-γ treated cells were the ones with the lowest arginase activity. Using a theoretically arbitrary-selected mean value of 15 U/L as a reference (estimated from Polat et al. [45]), we found that only IFN-γ Mo-Mφ yielded significantly lower arginase concentration (Table 1). For the following analysis, we used 15 U/L as the cut-off concentration to discriminate between low and high arginase-producing cells.

**Table 1.** Comparative statistical analysis of the arginase activity (U/L) in each group with respect to a theoretical value (15 U/L).

	LPS	IFN-γ	Azithromycin	IL-4
Theoretical mean	15	15	15	15
Actual mean	25.83	7.853	17.49	19.37
Std. Error	6.623	1.571	1.315	4.404
Discrepancy	−10.83	7.147	−2.49	−4.367
95% CI of discrepancy	−17.66 to 39.33	−13.91 to −0.3855	−3.170 to 8.150	−14.59 to 23.32
t, df	t = 1.636 df = 2	t = 4.548 df = 2	t = 1.893 df = 2	t = 0.9915 df = 2
*P* value (two tailed)	0.2435	0.0451	0.1989	0.426
Significant (alpha = 0.05)?	No	Yes	No	No

**Figure 2.**
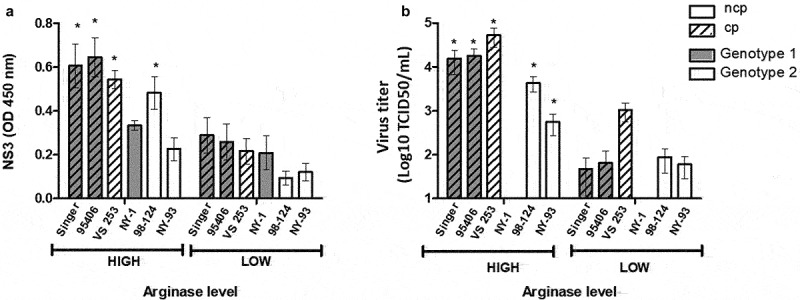
Replication of different BVDV strains in bovine macrophages with high or low arbitrary arginase activity levels. (a) NS3 antigen OD values; (b) virus titration in MDBK cells expressed as virus titre (Log10 TCID50/mL). Mean values ± SEM are depicted. *p < 0.05.

Infection was assessed by measuring intracellular expression of NS3 and by titrating infectious virus present in the supernatants. Values were categorized according to their arginase levels (low or high) defined as indicated above. In four of the six strains tested, NS3 values were higher in Mo-Mφ with high arginase levels compared to those with low levels (Figure 2a, *p* < 0.05). Differences in titres of newly produced virus were also significant between high and low arginase-producing Mo-Mφ for five strains. Only one strain (NY-1, Genotype 1b) did not produce viral particles, while NS3 expression was low but detectable (Figure 2b).

**Figure 3.**
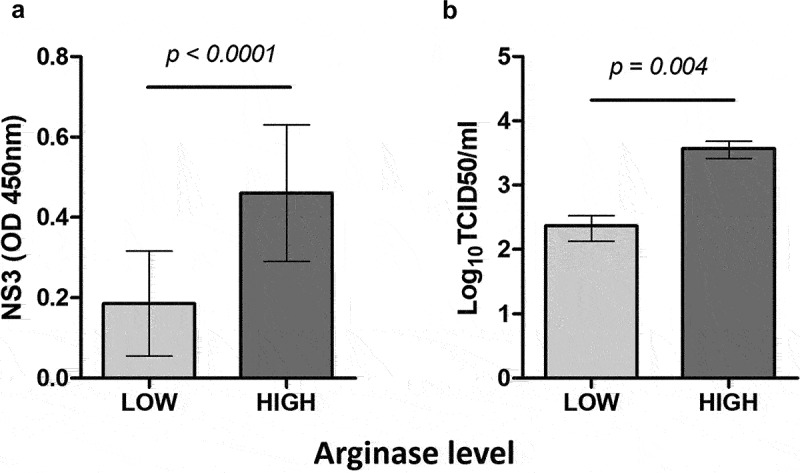
Association between arginase activity and infection by BVDV strains in Mo-Mφ with different arginase activity levels. Association between arginase activity and NS3 expression (a) or extracellular virus production (b) in Mo-Mφ stimulated with LPS, IFN-γ, IL-4 or azithromycin and infected with BVDV cp 1a Singer, 1b 95,409, 2a VS-253 and ncp strains 1b NY-1, 2b 98–124, 2 NY-93.

Mo-Mφ cultures were then grouped according to their arginase activity, irrespective of the treatments used to polarize the cells or the infecting virus strain. Cells with low arginase concentrations yielded low values in both NS3 level and viral titration assessments (Figure 3a and b, respectively). There was a significant association in Fisher Test between NS3 levels or viral titres with arginase activity (*p* < 0.0)). These results showed that viral replication was higher in Mo-Mφ with increased arginase concentrations, irrespective of the treatment they received or the viral strain used.

**Figure 4.**
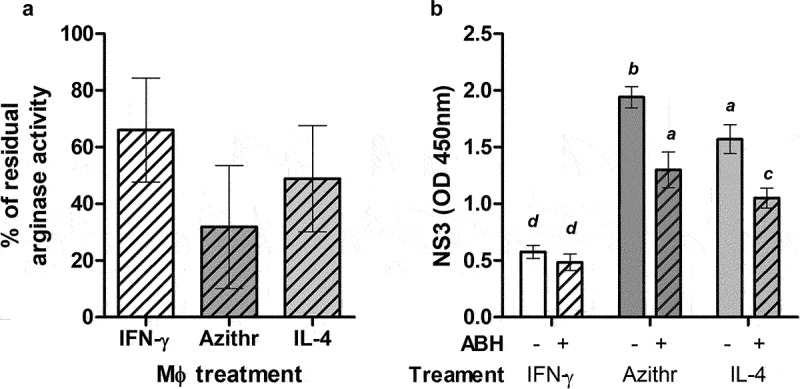
Inhibition of arginase activity in phenotyped bovine macrophages (a) and the effect of this inhibition in NS3 expression after infection (b). Monocyte-derived macrophages from four different steers were polarized and treated or not with 50 μM of ABH. Residual arginase activity (in percentage ± SD) is shown for each treatment. Mo-Mϕ treated or non-treated with ABH were infected with BVDV and NS3 expression was assessed using an antigen ELISA (mean OD values ± SD are shown). Differences in mean values were analysed using ANOVA followed by dunns with a confidence interval of 95%. Distinct letters over the bars indicate significant differences are present.

An experiment was designed to assess the impact of arginase inhibition on BVDV infection. Macrophages from four different BVDV-free animals were incubated with ABH (or mock-treated with RPMI medium) and cultured with azithromycin, IL-4, or INF-γ before infection. ABH treatment reduced arginase activity by 30% for INF-γ treated cells and between 60% and 80% for Mϕ treated with Azithromycin or IL-4, respectively (Figure 4a). No significant differences were found in arginase production in these cells after ABH treatment.

M1 and M2 Mϕ treated with ABH or mock-treated were infected with BVDV 98–124, and NS3 values were measured at 24 hpi (Figure 4b). Mo-Mφ treated with IFN-γ has the lowest NS3 levels, and these values were not reduced by ABH tratment. Significant higher replication rates were recorded for Mϕ treated with azithromycin, and IL-4 and NS3 levels were significantly reduced with ABH treatment in these cells (*p* < 0.05). Using ABH in these cultures significantly reduced NS3 levels in Mϕ treated with arginase-activity promoters, suggesting that BVDV replication is related to the arginase activity of these cells.

### Arginase activity of mo-Mφ from adult animals and calves

**Figure 5.**
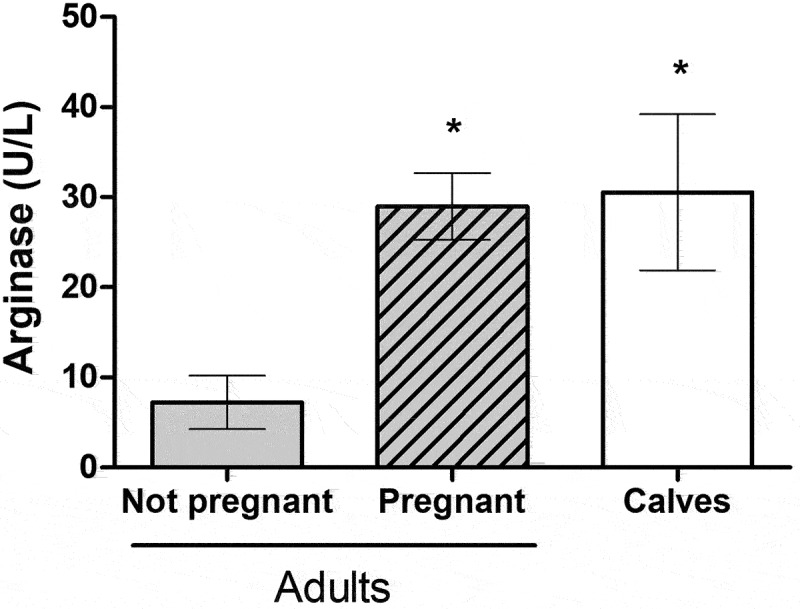
Arginase activity levels (mean ± SEM) in Mo-Mφ from animals with different physiological status. Mo-Mφ from adult cows (*n* = 4), cows in the first trimester of pregnancy (*n* = 4) and calves under 2 months of age (*n* = 3) were differentiated, and arginase activity was quantified. *: significant differences applying the one-way ANOVA test, followed by the Dunn´s test (*p* < 0.05).

BVDV infection is particularly successful in pregnant dams [11]. It is also well known from clinical practice that young animals have more evident BVDV symptoms than adults and that the disease lasts longer in young animals. Based on this knowledge, we evaluated the arginase levels of macrophages prepared from pregnant dams (*n* = 4), adult non-pregnant cows (*n* = 4), and 2-month-old calves (*n* = 3, Figure 5). Mo-Mφ from adult animals had significantly lower arginase activity than those from pregnant dams and calves that have similarly high levels of arginase (~30 U/L).

#### *Infection of* in vivo *stimulated mo-Mφ*

An experiment was designed to polarize macrophages towards a high-arginase M2 functional profile *in vivo*. Although there are reports of treatments that induce a certain profile of macrophages in the murine model [46–50], to the best of our knowledge no similar experiment has been reported in cattle. We hypothesize that the Azithromycin-treated animals would present greater replication in Mo-Mφ *ex vivo*, associated with higher arginase, typical of an M2 profile.

Six calves under 2 months of age were randomly allocated into two experimental groups of three animals each and named according to the treatment they received: Azithromycin and Not-treated (Control). The original experimental design contemplated the intravenous administration of 10 mg/Kg of azithromycin for four consecutive days [36]. One of the animals became stunned and disoriented after the IV azithromycin application. The symptoms subsided within a few seconds, and no veterinary intervention was required. Nonetheless and pursuing animal welfare, the administration was performed orally from then on (1 g per day). All the animals in the azithromycin group received the first dose IV on the first day, switching to the oral route for the following 3 d. The control group was not intervened.

The erythrocyte sedimentation rate, cholesterol, blood cells´ count, and liver-enzymes showed no differences between treatments or with reference values (Supplementary file 2) on day 0, 5 and 7 post-treatment. No significant differences were observed on serum arginase activity between groups (data not shown).

**Figure 6.**
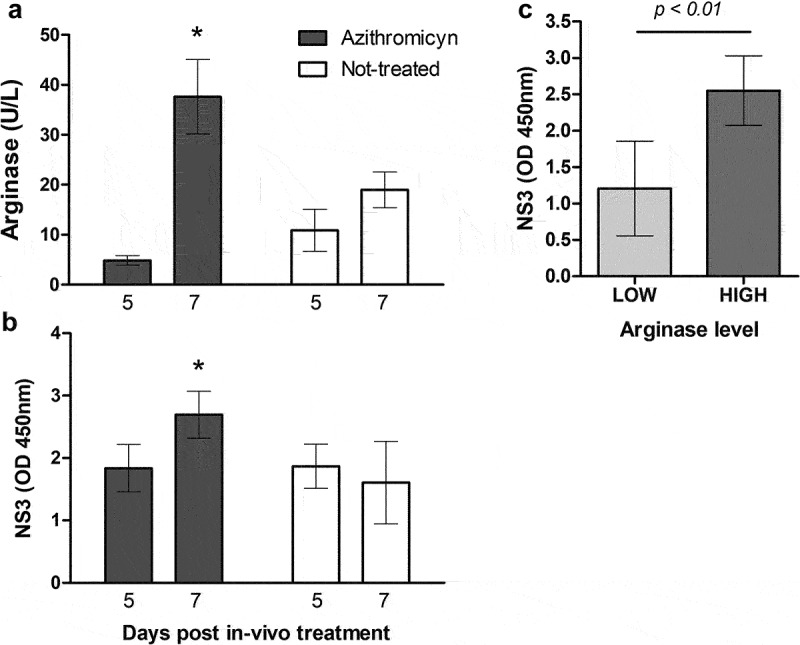
Intracellular viral replication and arginase activity in Mo-Mφ purified on days 5 and 7 post *in vivo* treatment with azithromycin and infected with BVDV 98–124 strain. (a) arginase activity levels in Mo-Mφ from the different animals before infection. (b) NS3 levels in Mo-Mφ infected with BVDV 98–124 at MOI = 2 (or mock-infected) at 24 hpi. (c) comparative NS3 levels respect to arginase activity. Mean values ± SEM are shown. Significant differences are indicated; * *p* < 0.05, compared to other days and values.

Mo-Mφ were obtained from peripheral blood on days 5 and 7 post-treatment. Arginase activity was considerably higher in Mo-Mφ from azithromycin treated animals purified on day 7 than in those obtained on day 5 (Figure 6a). There were no significant differences in arginase levels between day 5 and 7 in control animals. Arginase levels from Mo-Mφ azithromycin-treated animals were higher than those of control animals (*p* < 0.05) 7 d after treatment. No significant differences were found when arginase activity was compared between groups within day 5 (*p* > 0.05, Figure 6a).

Mo-Mφ derived from the azithromycin and control calves’ monocytes were infected with BVDV 98–124, a type-2 local strain that yielded high infection rate when Mo-Mφ were polarized *in vitro* (Figure 2). NS3 levels were significantly different for azithromycin-treated animals at 7 d, compared to all other values (*p* < 0.05). The general trend was for higher NS3 levels when arginase activity was high (Figure 6a and b), although no other significant differences were found between groups due to the high dispersion of the data (*p* > 0.05).

NS3 values were then analysed based on the arginase activity (assigned as high or low) regardless of the treatment the animals received and considering altogether data from 5- and 7-d post-treatment. The association between infectivity and Mo-Mφ arginase activity *in vitro*, described above, was verified after *in vivo* treatment, even though we had high dispersion of the analysed data. NS3 values in high arginase-producing Mo-Mφ were significantly higher than those from Mo-Mφ with low arginase activity (*p* < 0.01; Figure 6c). With this experiment, we identified a relationship between high arginase activity and BVDV infectivity using an *in vivo* treatment to polarize the macrophages.

## Discussion

This study provides the first evidence of the enhanced tropism of BVDV for arginase-producing, monocyte-derived bovine macrophages. Macrophages are polyfunctional cells with high heterogeneity and phenotypic plasticity that perform proinflammatory functions of eliminating pathogens and regulatory functions of restoring homoeostasis. In this context, the susceptibility to the virus of macrophages of certain physiological conditions may play a relevant role in the almost unexplored immunopathogenesis of this virus.

Arginase activity as a hallmark of the M2-spectrum macrophages´ profile is widely accepted [28,51–54]. The association between arginase activity and greater viral replication has been reported for viruses from various families [55] including the genus Pestivirus [56]. Different stimuli are used to induce M1 or M2 macrophages. We verified that both IL-4 and azithromycin induced higher levels of arginase than IFN-γ, as demonstrated before [40,50]. It was surprising to find that LPS-treated cells inclined Mo-Mφ towards an anti-inflammatory phenotype. Adherent-enriched cells were 90% positive for CD14, the LPS receptor (Supplementary file 1). Unpublished data from our laboratory show that LPS treatment induced either a pro or an anti-inflammatory state, which we attributed to each animal’s inflammatory background [37], as cells from the same animal prepared by positive CD14 selection or by differential adherence responded equally to LPS, either in a pro or anti-inflammatory bias. More studies are needed to understand the effect of LPS in this cell-type. Conversely, IFN-γ always induced low arginase levels irrespective of the animal or cell preparation (Supplementary file 1).

Gharib et al. highlighted the diversity of human macrophage polarization, attributed to functional consequences in response to different M2 stimuli [57]. Montoya et al. [58] made an exhaustive characterization of bovine Mo-Mφ and monocyte-derived dendritic cells. Mo differentiated with GM-CSF, IL-4, and FLt3L resulted in adherent and non-adherent cell cultures. Each adherent and non-adherent cell fraction was represented by a heterogeneous mixture of cells; however, there was no way to predict the proportion of cells of each type according to their adherence ability and these proportions were irreproducible between experiments, even when they were carried out with the same animal [58]. Therefore, due to the difficulties of working with bovine primary macrophage culture, we consider arginase activity as the most reliable marker to simplify the assessment of the interaction of BVDV with macrophages of different phenotypes.

Mo-Mφ treated with IFN-γ supported the least viral replication. These cells had low arginase activity, behaving as M1 cells according to the stimulus used. Conversely, azithromycin and IL-4-treated Mo-Mφ supported high viral replication with almost all the tested strains. Only one strain (BVDV NY-1) showed a limited replication in all Mo-Mφ cultures, and virus could not be detected from supernatants. This can be limited to this strain or related to genotype 1b [59]. More experiments are needed to verify these possibilities.

Infection with cytopathic strains caused massive death of IL-4-treated Mo-Mφ, while cells treated with azithromycin developed cytopathic effect, and metabolism was partially reduced compared to mock-infected cells. This difference in the performance of both M2-type cell preparation can be due to the different mechanisms that mediate the anti-inflammatory effect triggered by azithromycin and IL-4. These compounds elicit divergent transcriptional programs in human Mo-Mφ [57]. For instance, azithromycin anti-inflammatory mechanisms involve inhibition of the STAT1 and NF-κB signalling pathways through the drug’s effect on p65 nuclear translocation and IKKβ. Inhibition of NF-κB and STAT1 downregulate Toll-like receptor (TLR) 4 signalling [60]. Using an IKKβ inhibitor affects arginase production in azithromycin but not in IL-4 treated cells [40], meaning that the two molecules exert their function through distinct mechanisms and probably induce arginase-producing cells of different M2-state.

The association between arginase activity and virus infection was computed based on an arbitrary cut-off value, which can be arguable for a statistical analysis. To confirm these results, we performed and experiment inhibiting arginase with ABH. Mo-Mφ of the M2 profile treated with this arginase inhibitor were less infected by BVDV. These results suggest that not only M2 Mo-Mφ are more susceptible to BVDV but that also arginase activity might be involved in promoting infection. Fit-for-purpose experiments are needed to understand the mechanisms of BVDV replication in bovine Mo-Mφ. Additionally, considering that ABH is a compound with great therapeutic potential that has been evaluated in several disease models (sexual, immune, cardiovascular, and airway dysfunctions, reviewed by Clemente et al. [61]), our results pose focus on the possible role of ABH in the immuno-pathogenic outcome of those viral infection that could (as BVDV) have an enhanced infectivity due to or as a result of high arginase activity.

Arginase activity can have a profound effect on the development of the adaptive immune response. For instance, L-arginine depletion suppresses T-cell proliferation [62]. This may allow arginase-expressing M2 macrophages to dampen the CD4+ T-cell effector response and may worsen immunodeficiency. Viral-induced impairment of M1/M2 polarization favours the upregulation of Arginase. The distinct roles of M1 and M2 macrophages in BVDV infection raise the possibility that BVDV may promote M2 polarization of macrophages to impair the immune responsein particular, the Type 1 T helper cell immune response, resulting in persistent infection and disease progression. Fit-for-purpose experiments are needed to accept or dismiss this hypothesis.

The important role of macrophage phenotype on BVDV replication led us to analyse if there was a difference in the macrophage’s arginase activity between calves and adults and pregnant and non-pregnant animals. Purified Mo-Mφ from pregnant cows and calves had significantly higher arginase activity than that measured in Mo-Mφ from adult non-pregnant animals. This finding supports further exploring if arginase levels observed in the macrophages of the different age groups could be related to the greater susceptibility and severity of the infection in calves and pregnant females. It will also be interesting to explore if differences in the type of colostrum that the calves receive (i.e. whole colostrum containing maternal cells, pooled colostrum, or an artificial preparation) may affect the Mϕ profiles in calves. Pregnancy is a vulnerable period to BVDV infection, as the virus can cross the placental barrier generating the birth of PI calves. Neil et al. [11] demonstrated the high viral genetic variation that occurs during BVDV infection is particularly successful in pregnant dams. They hypothesized that this can be explained by “the tough-transit” model of Pfeiffer and Kirkegaard [63], which proposes that viruses have a difficult time passing the natural barriers and innate immunity of the host and the few viruses that succeed in bypassing the barriers establishing the new viral population. They also proposed a role of the altered immunity of these animals because of the pregnancy, allowing a greater number of infecting virus particles to establish a productive infection.

A higher prevalence of BVDV infections has been reported in calves [64]. On the other hand, the clinical manifestations in calves are more severe than in adults, especially associated with secondary bacterial infections [65]. It is also known from clinical practice that young animals evidence symptoms more frequently than adults and that the disease lasts longer. For viral diseases in general, young animals and the foetuses are vulnerable to infections by intracellular pathogens, like bacteria and helminths, and many of them induce and even express arginase [66]. Interestingly, neonates and young individuals usually have a higher proportion of Mφ and Mo of the M2 phenotype than adults [67]. This is an interesting point to further study.

There are several reports on azithromycin used as an inducer of the Mφ-M2 profile in the murine model [46–50], but there are no reports of using this antibiotic to promote the development of M2 macrophages in cattle. Azithromycin and other macrolide antibiotics have immunomodulatory properties and regulate inflammation *in vivo* [68]. This study provides the first demonstration of the capacity of azithromycin to induce arginase-producing Mo-Mφ of the M2 state in bovines. These *in vivo* polarized macrophages had high arginase activity and supported BVDV replication to higher rates than those from not-treated animals, confirming *in vitro* results. Arginase activity increased significantly from day 5 to 7 in azithromycin-treated calves, showing a growing trend. It would be interesting to sample at later time points after treatment and perform the experimental infection of these animals at the time of the Mo-Mφ highest arginase activity. It is remarkable that the use of an antibiotic can have such a profound effect on the calves' innate immune system. This concept deserves to be explored in greater depth to provide adequate recommendations for the use of this antibiotic in veterinary practice.

Altogether, our results verified for the first time that arginase-producing, alternatively activated bovine macrophages support enhanced BVDV replication *in vitro* and *ex vivo*. Primary-cultured Mo-Mφ from azithromycin-treated animals had higher replication of BVDV associated with an increased arginase activity on day 7 post-treatment compared to non-treated animals, constituting a promising model for the study of the Mφ phenotypes in cattle. Understanding the impact of the bovine macrophage phenotype in BVDV infection may constitute a first step to unveil a possible leading role of these cells in establishing an immune-suppressive state and promote further studies to dissect the role of macrophage phenotype, which might also be consequential of the animal’s physiological state, in the outcome of bovine viral diseases.

## Supplementary Material

Supplementary file 1_Barone et al 2023.pdf

Supplementary file 2.pdf

## Data Availability

The data supporting the findings of this study are available within the article and on request from the corresponding author.
