# Supplementary material for: Enhanced infectivity of bovine viral diarrhoea virus (BVDV) in arginase-producing bovine monocyte-derived macrophages
Source: Virulence. 2023 Nov 15;15(1):2283899. doi: 10.1080/21505594.2023.2283899 (PMC11756584; doi:10.1080/21505594.2023.2283899)
Supplement: Supplementary file 1_Barone et al 2023.pdf [file KVIR_A_2283899_SM0409.pdf]

Supplementary file 1: characterization of M1 and M2 macrophages prepared from bovine blood monocytes

Macrophages were prepared from blood monocytes (Mϕ-Mo) as described in the Materials and Methods section. They were assessed for viability and CD14 expression (Figure 1). These cultures were mock treated (media) or treated with different stimulus to get a classical-activated profile (INF-γ) or an alternatively activated phenotype (using IL-4). The polarized Mϕ-Mo were analyzed for expression of surface markers (figure 2A) ,TNF-α, IL-10 production, and arginase activity 96 hours after starting the treatment (Figure 2B). The level of expression of different parkers per cell by analyzed comparing the mean fluorescence intensity (IFM). The expression of the costimulatory molecule CD86 tended to increase in Mϕ-M1, although with considerable dispersion. Mϕ-Mo treated with IL-4 down-regulated CD14 and CD172a. No changes were observed for CD205 expression (Figure 2A). However, and due to the high dispersion of the results, none of these differences were statistically significant and were considered as tendencies.
The phagocytic capacity of M1 and M2 cells with live bacteria was assessed. The phagocytosis index was established as CFU (ml-1) per number of cells (Figure 2A). Mϕ-Mo treated with IL-4 showed increased phagocytosis ($p < 0.05$). Mϕ-Mo stimulated with IFN-γ induced a significantly higher amount of TNF-α (Figure 2C) and a significantly lower amount of IL-10 ($p < 0.05$ in both cases, Figure 2D). The arginase activity was significantly increased in the M2 profile compered to M1 ($p<0.05$, Figure 2E). For all the determinations, three independent experiments were performed.

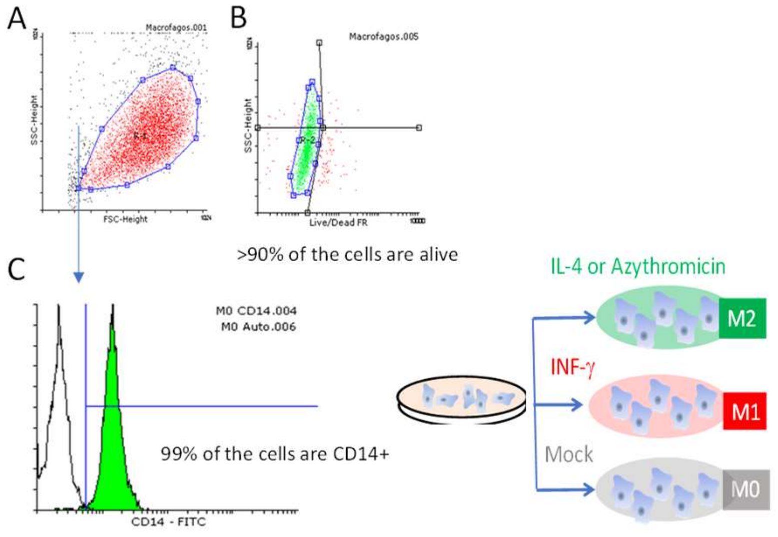

Figure 1 Flow cytometry analysis. Mϕ-Mo were purified by adherence (A), assessed for viability (B) and labelled with an anti-CD14-FITC antibody (c). Almost 99% of the Mϕ-Mo obtained by adherence were CD14+. These cells were then incubated with different stimulants to produce M1 or M2 macrophages as indicated

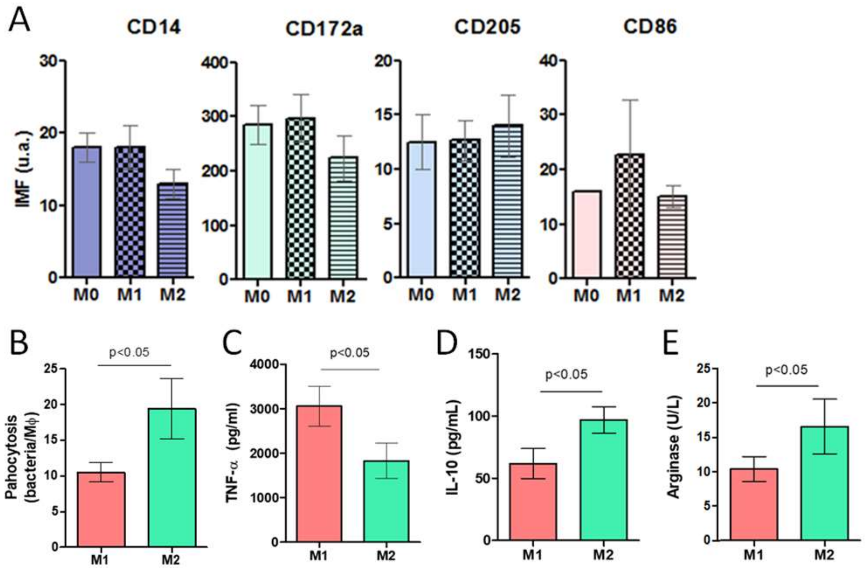

Figure 2 - Characterization of M1 and M2 macrophages. Monocyte-derived macrophages (Mϕ-Mo) were stimulated with IFN- (M1) and IL-4 or azithromycin (M2), or mock-treated for 24 hours (A)Surface markers were assessed using flow cytometry. MFI results for the different indicated markers are shown. (B) Mϕ-Mo were also analysed for their phagocytic capacity (measured using E.coli), for TNF-α and IL-10 production in culture supernatants (ELISA) and arginase. Experiments performed using blood from four different animals each time . Significant differences ($p < 0.05$) applying the Mann Whitney U test are indicated
