# Supplementary material for: Enhanced infectivity of bovine viral diarrhoea virus (BVDV) in arginase-producing bovine monocyte-derived macrophages
Source: Virulence. 2023 Nov 15;15(1):2283899. doi: 10.1080/21505594.2023.2283899 (PMC11756584; doi:10.1080/21505594.2023.2283899)
Supplement: Supplementary file 2.pdf [file KVIR_A_2283899_SM0408.pdf]

Supplementary file 2: Clinical hematology data of calves treated with oil adjuvant, azithromycin or not-treated (control).

A

Group	Animal	Day	ERI (mm)	PrCR (mg/dl)	COL (mg%)	BD (mg%)	BT (mg%)	TGO (UI/l)	TGP (UI/l)	FAL (UI/l)	PRO (g%)
Azithromycin	513	1	1	0,40	131	0,2	0,7	64	15	354	6,1
		5	1	0,42	137	0,2	0,5	58	13	296	6,1
		7	1	0,29	124	0,2	0,6	54	13	304	6,2
	517	1	1	0,73	151	0,2	0,7	66	13	380	5,9
		5	1	0,82	142	0,2	0,5	54	10	305	5,8
		7	1	0,10	100	0,2	0,7	53	10	332	5,9
	518	1	1	0,67	108	0,2	0,7	47	11	439	6,4
		5	1	0,84	124	0,2	0,6	45	8	411	6,6
		7	1	0,15	79	0,2	0,6	42	10	431	6,8
Oil Adjuvant	504	1	1	0,50	152	0,2	0,7	63	10	315	5,9
		5	1	0,41	131	0,2	0,7	62	10	317	5,6
		7	1	0,05	78	0,2	0,7	55	11	321	5,8
	505	1	1	0,28	108	0,2	0,7	66	15	187	5,7
		5	1	0,08	60	0,2	0,6	53	11	118	5,5
		7	1	0,23	88	0,2	0,6	49	11	148	5,8
	508	1	1	0,51	130	0,2	0,7	56	12	389	6,2
		5	1	0,22	94	0,2	0,7	60	12	287	6,3
		7	1	0,04	53	0,2	0,7	45	10	299	6,4
Control	506	1	1	0,40	107	0,2	0,7	66	22	101	6,3
		5	1	0,28	102	0,1	0,5	80	21	91	6,1
		7	1	0,04	65	0,1	0,5	60	17	90	6,2
	509	1	1	0,27	93	0,2	0,7	58	12	314	5,9
		5	1	0,13	77	0,2	0,6	55	10	259	5,7
		7	1	0,04	52	0,2	0,5	49	9	245	6,0
	514	1	1	0,29	112	0,2	0,7	54	13	178	5,6
		5	1	0,12	106	0,2	0,7	70	14	174	5,5
		7	1	0,03	54	0,1	0,4	47	12	148	5,5

B

Group	Animal	Day	Red blood cells (/mm^3)	Hto (%)	Hb (g/100 ml)	VCM (fl)	HCM (pg)	CHCM (g/dl)	RDW-C.V. (%)
Azithromycin	513	1	8500000	36,0	11,9	42,35	14,00	33,06	26,2
		5	8390000	34,9	11,7	41,60	13,95	33,52	26,0
		7	8940000	37,2	12,5	41,61	13,98	33,60	26,3

		1	7960000	34,6	11,1	43,47	13,94	32,08	23,6
	517	5	7460000	31,9	10,5	42,76	14,08	32,92	23,0
		7	8380000	35,5	11,9	42,36	14,20	33,52	24,2
		1	7280000	30,9	11,1	42,45	15,25	35,92	28,2
	518	5	7740000	32,8	11,9	42,38	15,37	36,28	28,2
		7	7840000	33,0	12,1	42,09	15,43	36,67	28,3
		1	7510000	30,4	11,5	40,48	15,31	37,83	28,6
	504	5	7000000	27,8	10,6	39,71	15,14	38,13	26,9
		7	6930000	27,2	10,7	39,25	15,44	39,34	27,1
		1	7600000	32,6	11,0	42,89	14,47	33,74	26,2
Oil Adjuvant	505	5	7290000	30,3	10,8	41,56	14,81	35,64	26,0
		7	7510000	31,0	11,1	41,28	14,78	35,81	26,4
		1	8630000	35,1	12,0	40,67	13,90	34,19	26,6
	508	5	8980000	36,3	12,8	40,42	14,25	35,26	26,7
		7	8740000	34,6	13,0	39,59	14,87	37,57	26,9
		1	7410000	27,9	11,2	37,65	15,11	40,14	24,9
	506	5	7400000	27,8	11,4	37,57	15,41	41,01	25,1
		7	7300000	27,2	11,5	37,26	15,75	42,28	25,2
		1	6570000	25,0	10,0	38,05	15,22	40,00	26,5
Control	509	5	6740000	25,7	10,3	38,13	15,28	40,08	26,2
		7	6680000	25,2	10,7	37,72	16,02	42,46	26,1
		1	7530000	32,4	10,9	43,03	14,48	33,64	28,5
	514	5	7590000	31,8	11,2	41,90	14,76	35,22	28,7
		7	7700000	31,9	11,4	41,43	14,81	35,74	29,0

C

Group	Animal	Days	Leucocytes (/mm3)	Band neutrophils (%)	Segmented neutrophiles. (%)	Eosinophils (%)	Basophiles (%)	Monocytes (%)	Lymphocytes (%)
		1	9100	0	28	0	1	13	58
	513	5	12000	0	23	0	1	19	57
		7	8500	0	16	0	1	17	66
		1	9600	0	23	0	0	9	68
Azithromycin	517	5	9200	0	20	0	1	9	70
		7	9500	0	23	0	1	7	69
		1	9800	0	25	0	1	14	60
	518	5	14500	0	35	0	1	12	52
		7	11100	0	28	0	1	13	58
Oil Adjuvant	504	1	11000	0	18	0	1	17	64
		5	9100	0	16	0	1	14	69

		7	9700	0	18	0	1	8	73
	505	1	13800	0	17	0	1	16	66
		5	14000	0	14	0	1	14	71
		7	13100	0	24	0	1	13	62
	508	1	7000	0	19	0	1	14	66
		5	7400	0	13	0	1	13	73
		7	7600	0	17	0	1	13	69
Control	506	1	12600	0	30	0	1	7	62
		5	9800	0	18	0	1	9	72
		7	11000	0	18	0	1	8	73
	509	1	11600	0	22	0	1	10	67
		5	8900	0	9	0	1	11	79
		7	10100	0	17	0	1	16	66
	514	1	6200	0	18	0	1	11	70
		5	6200	0	20	0	1	10	69
		7	7500	0	22	0	2	12	64

Clinical hematological data of calves treated with oil adjuvant, azithromycin or not-treated (control). Values correspond to days 1, 5 and 7 post -treatment. (**A**) erythrocyte sedimentation rate (ERI), C-reactive protein (PrCR), cholesterol (COL), direct bilirubinemia (BD), total bilirubinemia (BT), glutamic oxalemia transaminase (TGO). Pyruvic glutamic acid (TGP), alkaline phosphatase (ALP), serum proteins (PRO). (**B**) Hemogram: red blood cells, hematocrit (Hto), hemoglobin (Hb), mean corpuscular volume (MCV), mean corpuscular Hb (MCH), mean corpuscular Hb concentration (CHCM), erythrocyte distribution width (RDW-C.V.). (**C**) Immune cell counts.
